# Achilles Tendinopathy Pathogenesis and Management: A Narrative Review †

**DOI:** 10.3390/ijerph20176681

**Published:** 2023-08-30

**Authors:** Domiziano Tarantino, Rosita Mottola, Giuseppina Resta, Rossana Gnasso, Stefano Palermi, Bruno Corrado, Felice Sirico, Carlo Ruosi, Rocco Aicale

**Affiliations:** 1 Department of Public Health, University of Naples Federico II, 80131 Naples, Italy; rosi.mottola@studenti.unina.it (R.M.); rossana.gnasso@unina.it (R.G.); stefano.palermi@unina.it (S.P.); bruno.corrado@unina.it (B.C.); felice.sirico2@unina.it (F.S.); caruosi@unina.it (C.R.); 2 Department of Orthopaedic and Trauma Surgery, Casa di Cura di Bernardini, 74121 Taranto, Italy; giusyresta96@gmail.com; 3 Department of Musculoskeletal Disorders, Faculty of Medicine and Surgery, University of Salerno, 84084 Baronissi, Italy; r.aicale@studenti.unisa.it

**Keywords:** Achilles tendon, Achilles tendinopathy, tendinopathy

## Abstract

The Achilles tendon is the thickest and strongest tendon of the human body, and it is frequently injured during sports activity. The incidence of Achilles tendon pathologies has increased over recent decades, especially in the last few years, because of increased sports participation among the general population and due to the diffusion of competitive sports at a high level. Tendinopathies are common in athletes and in middle-aged overweight patients. The term “tendinopathy” refers to a condition characterised clinically by pain and swelling, with functional limitations of tendon and nearby structures, and consequently to chronic failure of healing response process. Tendinopathies can produce marked morbidity, and at present, scientifically validated management modalities are limited. Despite the constantly increasing interest and number of studies about Achilles tendinopathy (AT), there is still not a consensual point of view on which is the best treatment, and its management is still controversial. AT can be treated conservatively primarily, with acceptable results and clinical outcomes. When this approach fails, surgery should be considered. Several surgical procedures have been described for both conditions with a relatively high rate of success with few complications and the decision for treatment in patients with AT should be tailored on patient’s needs and level of activity. The aim of this article is to give insights about the pathogenesis and most used and recent treatment options for AT.

## 1. Introduction

The Achilles tendon is the strongest and largest tendon in the human body, and it can be affected by both degenerative and traumatic processes [1,2].

Tendinopathies are one of the most common orthopaedic diseases [3], and the Achilles tendon is the most frequently involved tendon, accounting for 20% of all tendon injuries [4,5].

Achilles tendinopathy (AT) is characterised by pain and swelling in and around the tendon, mainly arising from overuse, and it is a cause of disability to several athletes [6], especially those participating in track and field sports, volleyball, and soccer [7,8,9]; additionally, AT is also present in middle-aged overweight people and not physical active [8,10,11,12]. Its incidence remains unclear, due to a lack of epidemiological data [13]. The incidence and lifetime prevalence of AT in athletes is stressed by repetitive impact activities, i.e., running or jumping range from 9% to 52%, respectively [14,15].

Two types of AT have been described: insertional and non-insertional (depending on the affected site), characterised by different pathophysiology and treatment options [6,16,17]. Pain generation remains unknow [18] occurring at the beginning and after a training session, but as pathologic process progresses, pain may occur during the entire exercise session, interfering in daily living activities [19].

A proper clinical examination is the cornerstone of AT’s diagnosis, and imaging techniques can be useful as a support providing additional clinical information [20]. Clinically, pain is, generally, reported at 2 to 6 cm above the tendon insertion into the calcaneus [21].

**Figure 1:**
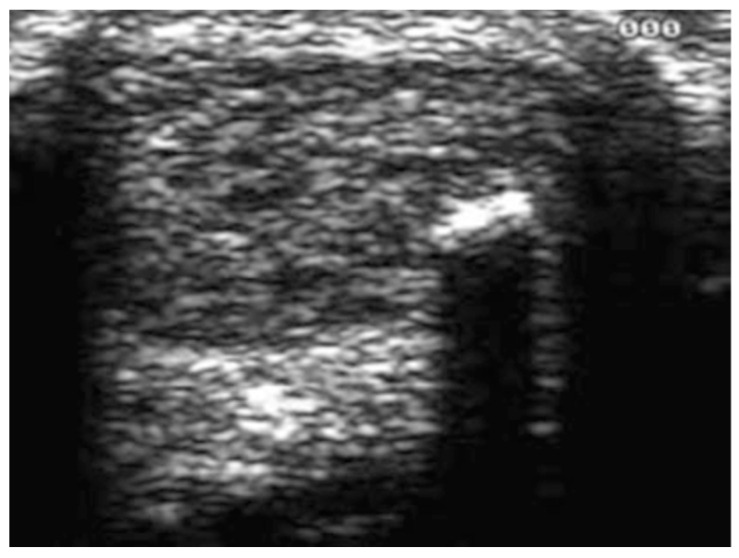
Ultrasonographic appearance of Achilles tendinopathy in a 28-year-old male soccer player at presentation. The longitudinal scan shows that the tendinopathic tendon is thicker than the asymptomatic contralateral one. The normal, well-ordered fibril distribution is lost.

Conservative AT treatments lack strong evidence support, and affected patients have an increased risk of long-term morbidity with undefined clinical outcomes [22]. Diagnostic imaging, such as plain radiography, ultrasounds (US) (Figure 1) and magnetic resonance imaging (MRI), can be required to verify or to exclude a clinical suspicion [23].

Treatment options for AT are primarily conservative (i.e., nonsteroidal anti-inflammatory drugs (NSAIDs), eccentric exercises (EE), low-energy shockwave therapy, etc.) [20,24], while surgery (open or minimally invasive) is indicated when the conservative treatment fails [1]. Growing evidence supports the use of biological therapies for the treatment of tendinopathies [25].

The aim of this narrative review is to give readers comprehensive knowledge of the pathogenesis and management of AT.

## 2. Methods

All the procedures related to this review were organised and reported after performing a search in the main scientific electronic databases (PubMed, Scopus and Web of Science) to identify the available scientific articles about the pathogenesis and management of AT, with no restrictions of time and language.

For the purposes of our review, we used several combinations of the following keywords: Achilles tendinopathy, Achilles tendinopathy pathogenesis, Achilles tendinopathy management, Achilles tendinopathy treatment, etc, in combination or using Boolean operators, such as “Achilles” AND “tendinopathy” AND (“management” OR “treatment”).

All kind of articles, such as systematic reviews and meta-analyses; randomised clinical trials (RCTs); and prospective, retrospective and case-series studies were included to give readers the most comprehensive overview about AT’s pathogenesis and management.

## 3. Discussion

### 3.1. Pathogenesis

#### 3.1.1. Clinical Examination

Clinical examination is essential for a correct diagnosis and management of AT, which typically occurs with pain 2–6 cm proximal to the tendon insertion, at the beginning; after exercise; and, then, with the progresses of the pathological process, during exercise, interfering with activities of daily living in severe cases [22]. For example, runners experience pain at the beginning and the end of training, with diminished discomfort in between [22]. The degree of morning stiffness is directly correlated with the severity of the disease.

Examination begins with explosion of both legs, with the patient in the standing and prone positions. Malalignment, deformity, asymmetry in tendon size and localised thickening need to be evaluated. The Achilles tendon should be palpated to detect tenderness, heat, thickening, nodularity and crepitation [26].

The “painful arc” sign better indicates tendinopathy than paratendonitis [27], while the Royal London Hospital (RLH) test indicates AT if the pain evoked palpating the tendon with the ankle in neutral position disappears or is reduced when the tendon is palpated with the ankle in active dorsiflexion [21]. Maffulli et al. reported no difference in sensitivity, specificity, reproducibility, and predictive value of the painful arc sign and RLH test in the case of AT; however, when the tests were combined, the overall sensitivity and specificity increase [28].

Hutchinson et al., in 2013, found that two tests (location of pain and pain to palpation) are sufficient and accurate for clinical use [21].

#### 3.1.2. Risk Factors

Systemic comorbidities can be a risk factor for tendon injuries, affecting their structures directly or due to the systemic alteration of the local growth environment [29].

Diabetes mellitus is associated with tendinopathy altering cellular metabolism, increasing the intracellular content of water leading to oedema; decreasing the ability to tolerate ischemic stress; and increasing cross-links within collagen fibres due to advanced glycosylation end-products, altering their structure.

Collagen fibrils disorganisation can be also due to dyslipidaemia, decreasing their density, or for hypercholesterolemia, an abnormal fat deposition can be macroscopically seen in tendons, forming xanthomas.

Inflammatory arthropathies, such as rheumatoid and psoriatic arthritis, can cause direct tendon damage and inflammation, altering healing process.

Several genetic factors may play a major role in tendon strength and capacity to recover after an injury. Variations in genetic loci have been retrieved as part of distinct clinical disorders [30].

Intrinsic factors directly affect tendon health and composition in different ways; age, body mass index (BMI), nutrition, metabolic diseases, foot alteration such as pes cavus, forefoot varus, ankle lateral instability, limited flexibility and muscle weakness have been all recognised as possible risk factors [31].

Extrinsic factors influence tendon from the outside. The most important are excessive loading and fatigue loading, repetitive loading over the physiological range that can cause microtraumas, improper loading (for example, due to incorrect sport technique and/or inappropriate equipment) and disuse (even with no physiological stress levels, tendons can degenerate and decrease their mechanical properties) [32]. Furthermore, exogenous damage, which occur from sources outside of the body, need to always be evaluated, such as smoking or a direct mechanical injury. Fluoroquinolones and corticosteroids have been implicated as risk factors in tendinopathy [33]. The former enhances interleukin-1β-mediated MMP3 release; inhibits tenocyte proliferation; and, as corticosteroids, reduces collagen and matrix synthesis [34,35].

#### 3.1.3. Mechanisms of Injury

Over 50% of tendon injuries happen during sport activity in an overload acting and repetitive microtrauma overcoming physiological limits [36]. Subclinical damages remain occult and can accumulate before pain onset. Tendon tears are commonly classified as acute or chronic with intrinsic or extrinsic factors risk factors. In acute injury, extrinsic factors predominate [37]. The healing process responses to injured tendon evolve following three overlapping phases characterised by distinctive cellular and molecular process: inflammation, proliferation and remodelling [38,39]. However, repaired tissue is a fibrous scar and will never completely regain its pre-injury structural and functional properties [40].

Regarding chronic injury, despite the repair process being similar to acute ones, following microscopic and biomechanical animal studies [41], neo-tissue is weaker than uninjured tissue due to poorly aligned immature collagen fibrils [42]. For this reason, the chronically injured tendon might benefit from surgery [38,43].

#### 3.1.4. Histological Characterisation of AT

Tendinopathy can be seen as a failure in the adaptation of the cell matrix to different stresses because of an imbalance between matrix degradation and synthesis [44]. Microscopically, affected tendons have lost their normal glistening-white appearance, becoming grey-brown and amorphous. At the beginning, tendinopathic changes are often silent, being manifest directly with rupture, or coexist with symptomatic paratendinopathy [45].

**Figure 2:**
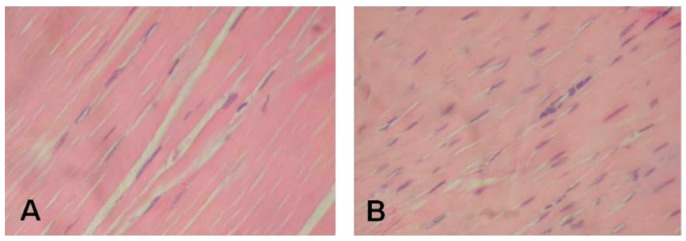
Rat tendon histology (H&E 40×). (**A**) Longitudinal section of a healthy Achilles tendon showing normal parallel orientation of the collagen fibres and presence of tenocytes with characteristic elongated nuclei. (**B**) Longitudinal section Achilles tendon 10 days after collagenase treatment showing obvious changes in the orientation of the collagen fibres, increased tenocyte number with roundness of their nuclei. This figure was retrieved from the following article: Autologous Leukocyte-Reduced Platelet-Rich Plasma Therapy for Achilles Tendinopathy Induced by Collagenase in a Rabbit Model [47]. This article is licensed under a Creative Commons Attribution 4.0 International License (https://creativecommons.org/licenses/by/4.0/ accessed on the 28 July 2023), which permits unrestricted use, distribution, adapting and reproduction in any medium, including images or other third party material. The original figure (Figure 4 in the original article) was changed by including only parts A and B and limiting the description of the figure to the lines related to parts A and B. The heading was changed too.

Histological alterations seen in tendinopathy are the non-inflammatory intratendinous collagen fibre degeneration, with an alteration in orientation and a reduction in fibre thickness and vascular ingrowth, an increase in type-III collagen and interfibrillar glycosaminoglycans production [31], modification in the cell density (i.e., presence of lots rounded tenocytes area, that seems to be chondroid cells, and other areas with a smaller amount of tenocytes), an increase in extracellular matrix (ECM) and poor healing response without inflammatory signs [46] (Figure 2).

### 3.2. Management Options

The management of AT largely lacks evidence-based support, and patients are at risk of long-term morbidity with unpredictable clinical outcomes [22]. The initial management is conservative, with many patients showing satisfying outcomes, but when it fails, surgery is recommended, usually after at least six months of conservative management [1,48,49].

### 3.3. Conservative Treatment

During the last years, several non-operative treatments have been proposed, such as the local drug injections (for example, corticosteroids, high-volume image-guided injections ((HVIGI) and physical therapy (i.e., shockwave or ultrasound therapy))), assuming a constantly increasing important role. However, most of the conservative treatments still lack solid scientific evidence [17].

#### 3.3.1. Pharmacological Interventions

NSAIDs are used for AT management, but data showed only a modest effect on acute symptoms for the short term [50], and even though tendinopathies are not considered a classical inflammatory condition [51], they can be useful for pain control to permit patients a correct eccentric strengthening performance, as well as gastrocnemius and soleus stretching. The potential negative effects of NSAIDS (such as ulcers, hypertension, etc.) need to be taken into consideration for each patient [24]. Furthermore, this analgesic effect may lead patients to ignore early symptoms, increasing the risk of further damage to the affected tendon, delaying good healing.

Cryotherapy, widely used for analgesia, reduces tendon metabolic rate, decreasing blood and protein extravasation from new capillaries retrieved in tendon injuries [52]. However, no evidence that this can be considered an effective treatment for AT has been found [20,24].

The efficacy of nitric oxide subministration via an adhesive patch in patients with mid-portion AT was evaluated by Paoloni et al. [53]. Topical glyceryl trinitrate is effective in the case of chronic non-insertional AT, with the benefits lasting for three years [54]. However, more recent studies questioned its mentioned benefits [55], and a recent systematic review and meta-analysis found no evidence that topical glycerin trinitrate is more effective at reducing pain in AT than the placebo [56].

#### 3.3.2. Therapeutic Exercise

Exercise programs consisting of both eccentric and concentric exercises are considered the keystone of the conservative management of AT, and they are widely used as a first-line treatment [18], with better results compared with wait-and-see treatment [57,58]. Eccentric exercises were shown to promote collagen fibres’ cross-link formation within the tendon, thereby facilitating tendon remodelling [16,59].

**Figure 3:**
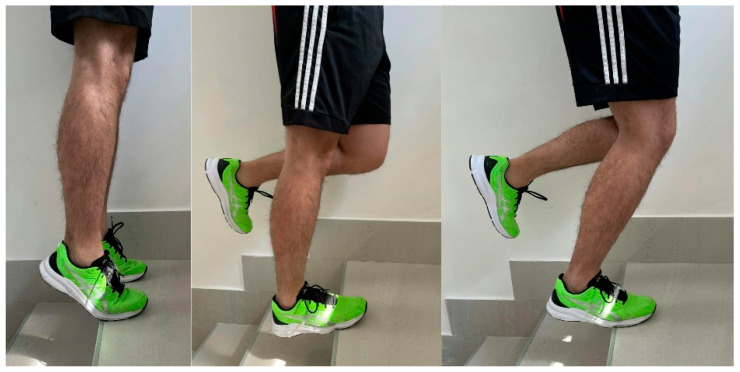
Eccentric loading exercises from Alfredson’s protocol for right mid-portion AT.

EE are the most effective conservative treatment for non-insertional AT [60,61,62], with good results reported [19,63] using the Alfredson’s protocol (Figure 3), which seems to be the most used protocol. Exercises need to be performed in three sets of 15 repetitions, two times per day, 7 days a week for 12 weeks [64].

A recent meta-analysis reported that most of the studies adopted Alfredson’s protocol [65]. Alfredson and other Scandinavian authors have reported excellent results in prospective randomised control trials [66,67,68].

The results of eccentric training proposed by other studies are less satisfying, with 50–60% good outcomes both in athletic and sedentary patients [48] or even lower [48,69]; these results may be influenced by many factors such as the motivation and compliance of patients.

Eccentric exercises alone may not work in all patients [48], and the mechanism of action is not completely understood [19]. However, the overall trend suggests a positive effect of EE in the complete absence of adverse effects [59].

The combination of eccentric training and shock wave therapy showed higher success rates when compared with these treatment modalities alone [70,71].

Eccentric–concentric progressing to eccentric (i.e., Silbernagel combined) [68] and eccentric–concentric (i.e., Stanish and Curwin) [72] have been studied [60], and a recent systematic review reported equivalent results to those of the traditional Alfredson’s protocol [62,73] when they were combined.

Both eccentric and concentric exercises could be considered as equally good, but given the lack of high-quality studies, no firm conclusions can be made on relieving pain, improving function or patient satisfaction [18].

Isotonic, isokinetic, and concentric loadings have also been described, showing inferior outcomes compared with the eccentric-type exercises [66,74].

Another kind of therapeutic exercise for AT is the heavy slow resistance training, which was found to be equally clinically effective as EE, but with a trend to be associated with greater patient satisfaction after 12 weeks compared with EE [75,76].

Recent evidence showed that strength deficits in triceps surae activity in patients with AT in the affected limb can be retrieved [55]. Probably, a reduction in load magnitude, rather than the loading rate, within the Achilles tendon can be more important to achieving a symptomatic benefit for AT [77]. A load-reducing effect due to an in-shoe orthotic heel lift can be used for AT management to reduce muscle activity in triceps surae. However, its use combined with footwear that incorporates a positive heel offset can be useful in a progressive-loading intervention program [78], especially for mid-portion AT [79].

#### 3.3.3. Physical Therapy

Where available, extracorporeal shockwave therapy (ESWT) can be used as a second-line option of management [1,36,60,80]. It acts on two different aspects of clinical response: tissue healing and pain transmission [60,81,82].

ESWT generates high strains in tendon, producing an analgesic effect and stimulating the tissue healing response [83,84,85]: additionally, there are no consensus regarding their application method, generation, energy level, number, and treatment frequency [86,87].

Several studies showed that low-energy ESWT when compared with EE is more effective than EE alone for insertional AT [88] but equal in the case of mid-portion AT at short term follow-up.

The combined use of ESWT and EE was found to be beneficial and may produce superior outcomes to EE alone in mid-portion AT [70,71].

However, when low-energy ESWT is not used following the recommendations and modalities outlined in the available scientific literature, the results could be poor [89].

Moreover, a recent systematic review and meta-analysis showed no general greater advantage of physical therapy than EE in the treatment of chronic AT [90].

**Figure 4:**
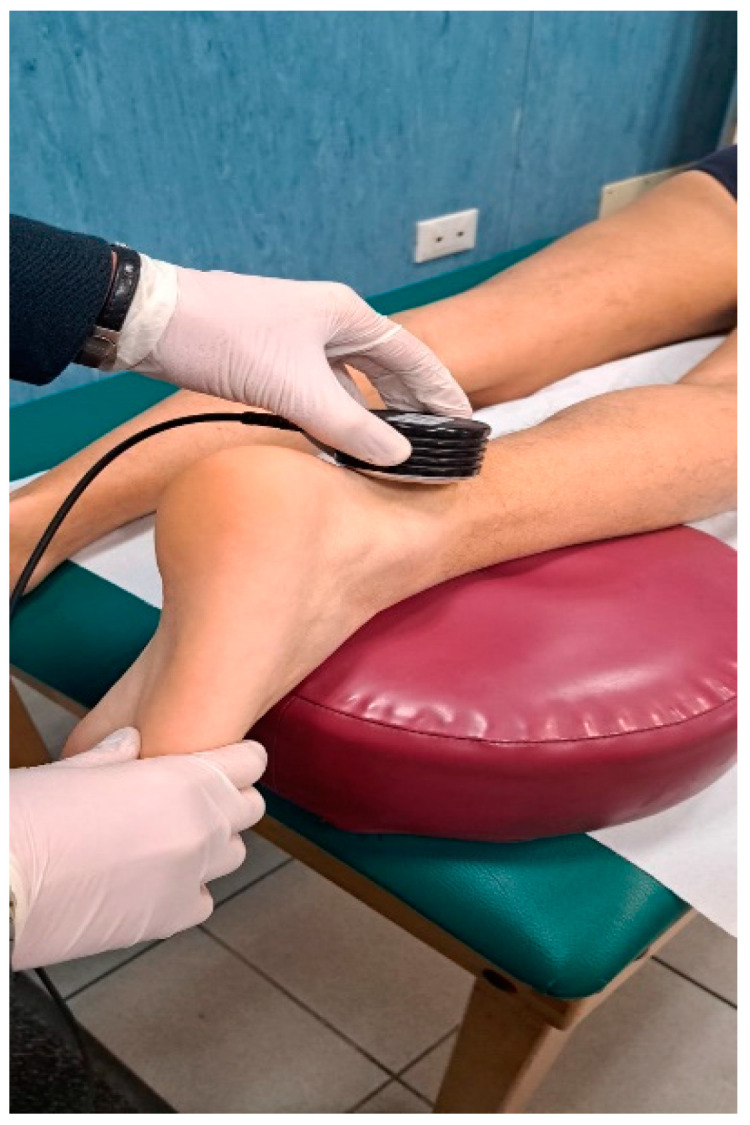
Ultrasound therapy for mid-portion AT.

Ultrasound (US) therapy is widely available and frequently used [24] (Figure 4).

Different studies concluded that insufficient evidence has been provided to support the beneficial effect of US therapy at the currently applied clinical dosages [91]. A RCT by Chester et al. found similar results between heavy eccentric loading and US therapy for treatment of AT in patients with a relatively sedentary lifestyle, with no adverse effects [92]. A recent retrospective observational study by Agostini et al. [93] showed that the simultaneous delivery of cryotherapy and ultrasound therapy is beneficial in patients suffering from AT, representing a good possibility of synergistically exploiting both therapeutic actions.

Hyperthermia may be considered an alternative therapeutic option to manage patients with AT, and some studies confirm these potential advantages [94].

#### 3.3.4. Orthotics

Orthotics are used for conservative treatment, but little evidence supports their use [95]. In patients with chronic Achilles tendon pain, no significant differences between the use of AirHeel^®^ brace and an eccentric training protocol were reported [62,96]: furthermore, a combination of eccentric training and AirHeel^®^ brace does not produce a synergistic effect [62,96,97,98].

#### 3.3.5. Injection Therapy

Various injection therapies have been used for AT [24,99]. Actually, studies that demonstrate the superiority of one injection technique or of one drug over another are few [24].

The use of platelet-rich plasma (PRP) for the management of AT is growing exponentially and it has been proposed as a second-line treatment [61], but only one RCT published about the effectiveness of PRP in AT and showed no significant difference in pain or activity level between PRP and saline injection at 6, 12 or 24 weeks when combined with EE [100]. Recent systematic reviews and meta-analyses all agree that, even if a trend towards pain reduction and functional improvement from baseline was observed after ultrasound-guided PRP injections, PRP was not more effective than a placebo (sham injection, no injection or physiotherapy alone) [101,102,103,104,105].

Furthermore, no standards for PRP dosage, injection technique, timing or number of injections are validated [106].

**Figure 5:**
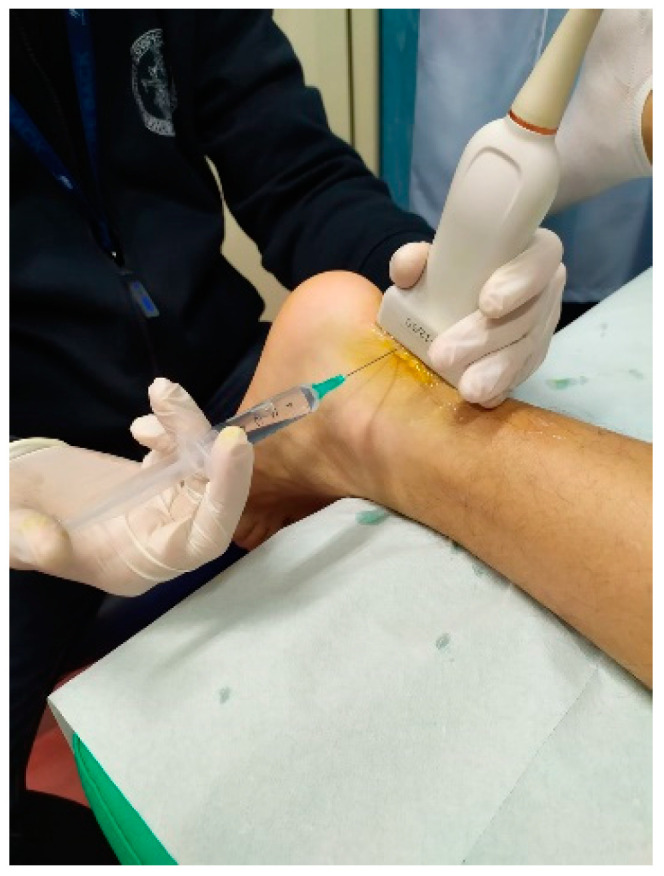
HVIGI for mid-portion AT.

Sclerosing injections showed contrasting results [18,107], with HVIGI producing local mechanical effects, from the stretching, breaking or occlusion of vessels and nerves arising during failure tendon healing response to reducing pain and improving function in patients with resistant AT [13,108] (Figure 5).

HVIGI are also able to be more effective, improving the results of chronic AT compared with PRP in the short term [109]. However, it is not possible to draw firm, evidence-based conclusions on the effectiveness of the different substances investigated (such as normal saline, corticosteroids, and local anaesthetics) [18].

Recently, the use of peritendinous injections of hyaluronic acid (HA) has been proposed for the treatment of AT [110] and resulted in being safe and well-tolerated, improving pain and function along with ameliorations in tendon structure and neovascularisation [111,112,113,114]. The use of HA injections with respect to ESWT for AT showed a significantly benefit with HA for pain relief with compared with ESWT, with a decrease of 68.1% versus 47.9% at the 4-week follow-up and score improvements at three and six months [115].

However, most of these results come from low-level studies, so further high-quality research is needed to confirm these promising outcomes [116].

### 3.4. Surgical Treatment

Conservative management is unsuccessful in 24% to 45.5% of patients, and surgery is recommended, generally, after six months of non-operative treatment [117,118]. However, long-standing tendinopathies are associated with poor postoperative results and a greater rate of reoperation to reach an acceptable outcome [119].

Recently, a systematic review analysed the outcomes of four different surgical procedures (paratenon stripped or not, open tenotomy with longitudinal tenotomy, open tenotomy with removal of abnormal tissue and percutaneous longitudinal tenotomy), resulting in satisfactory results in more than 70% of cases for each surgical procedure: however, the authors concluded that these results with relatively high rates are not observed in clinical practice [120].

#### 3.4.1. Open Surgery for AT

**Figure 6:**
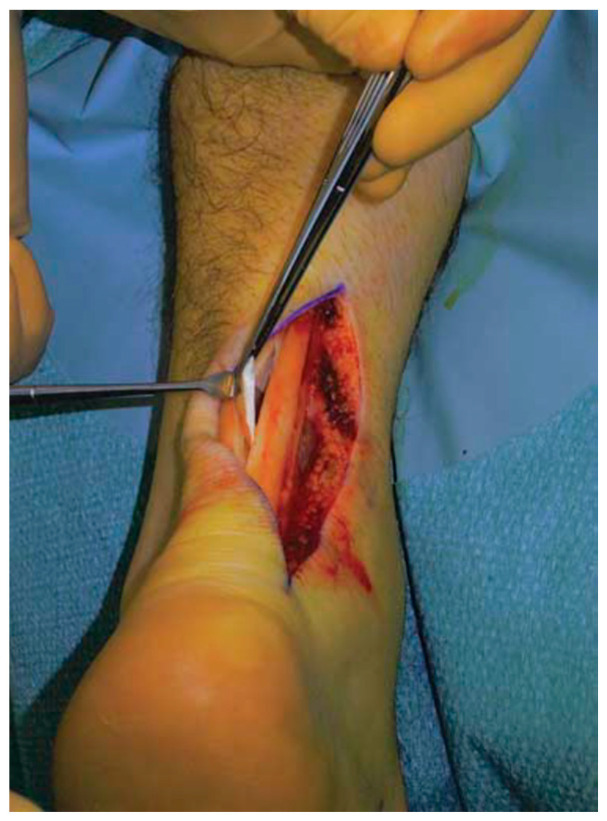
Open surgery for tendinopathy of the main body of the Achilles tendon. The tendinopathic tissue is identified and then excised.

Good results have been reported for open surgery, with a success rate between 50% and 100% [121,122,123,124], removing the intra-tendinous lesions and more than 50% of tendon debrided (Figure 6).

**Figure 7:**
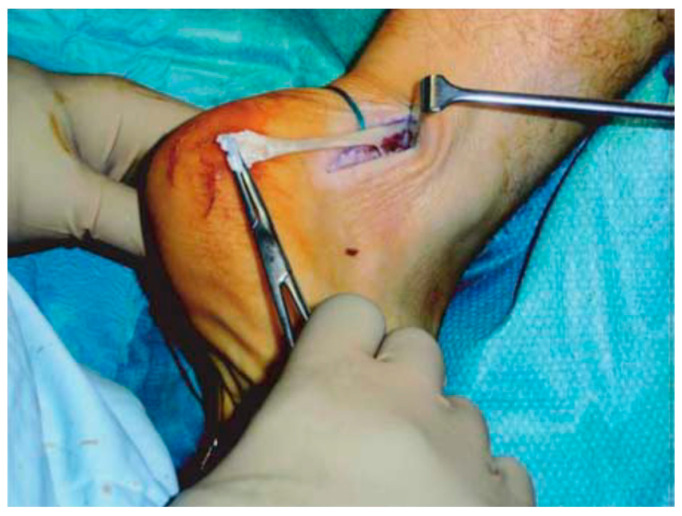
Photograph showing the peroneus brevis tendon being mobilised and pulled distally after the removal of adhesions and surrounding fibrosis.

**Figure 8:**
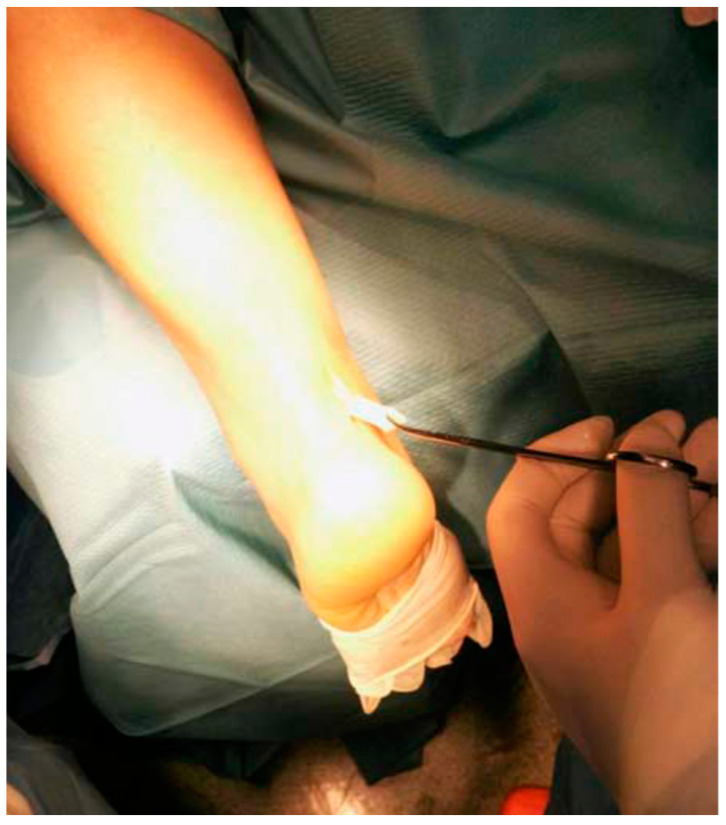
Flexor hallucis longus tendon graft was harvested through a 2.5 to 3 cm longitudinal medial incision along the distal portion of the Achilles tendon.

Longitudinal incisions are made from tendon medial size avoiding sural nerve and short saphenous vein injuries [18]. Tendinopathic tissue is identified and removed, and it appear with a disorganised fibre bundles with a “crabmeat” appearance [18]. Any gap can be repaired using a side-to-side repair and can remain unsutured, or if significant loss tissue occurs, a tendon augmentation or transfer can be considered. Peroneus brevis (Figure 7) or flexor hallux longus (Figure 8) tendons are the most frequently used local tendon grafts [43,125,126].

Then, early rehabilitation with early motion is advocated, avoiding tendon overloading during the initial healing phase [18].

The main preoccupation is the complication risk [61]; indeed, a study on 432 patients reported a complication rate of 11% [127], i.e., skin necrosis, wound infection, seroma formation, haematoma, cheloid scar formation, sural nerve apraxia, tendon rupture and thromboembolic disease.

For these reasons, patients should need to be informed regarding the procedure; their risks and complications; and at times, the prolonged recovery time [128].

#### 3.4.2. Minimally Invasive Techniques for AT

**Figure 9:**
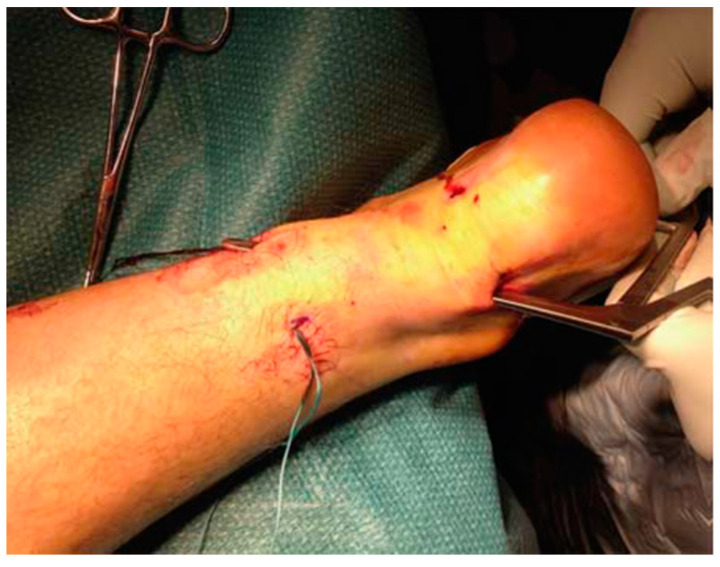
Minimally invasive percutaneous stripping for chronic Achilles tendinopathy. The four small incisions are visible, with the surgical instruments passing through.

A recent minimally invasive technique has been described with the aim to strip the paratenon from the tendon directly [6] or indirectly with high-volume fluid injection [108], reporting good results in relieving symptoms of non-insertional AT [129,130] (Figure 9).

Multiple percutaneous longitudinal tenotomies, performed under US guidance, produce good results, with the further advantage of being performed under local anaesthesia in an outpatient setting [131,132]. This surgical procedure can be performed in patients with isolated tendinopathy with no involvement of the well-defined paratenon and nodular lesion, less than 2.5 cm in diameter [131]. In the absence of chronic paratendinopathy, this procedure showed similar outcomes to those of open techniques [18]. Active dorsiflexion and plantar flexion of the foot are encouraged early after surgery [19].

In patients with chronic AT, the stripping of the neovessels inside the Kager’s fat triangle can be considered another kind of minimally invasive surgical treatment [18,19]. This technique permits a safe and secure breaking of neovessels and the accompanying nerve supply decreasing pain [18].

Good results were reported in the case of minimally invasive open debridement, with plantaris tendon resection showing minimal complications in elite athletes and patients with non-insertional AT [129,133,134,135,136,137].

Whatever the chosen treatment, patients are encouraged to weight bear as soon as possible after surgery [18].

Endoscopic procedures are also performed and are useful in minimising soft tissue damage and in supplementing junction without restoring anatomical continuity [24,43,60,126,127].

These procedures can be performed securely, resulting in good outcomes, and are effective, safe with low morbidity rate and less risks of infection, given their minimally invasive nature, allowing patients to return to pre-injury sport levels and daily activities [43,130]. Furthermore, they are technically easy to master and inexpensive [1].

In the state of the art, no studies compared the different minimally invasive procedures, and it is unclear when it is necessary to perform longitudinal tenotomies or to excise the plantaris tendon [60].

A recent review reported average success rates for minimally invasive techniques of 83.6% and 78.9%, respectively, and complication rates of 5.3% and 10.5%, respectively, compared with open procedures [122].

Therefore, a minimally invasive surgical approach for AT can be considered as an effective treatment option in the case of failure for conservative treatments, without the need to immediately resort to open surgery [130].

### 3.5. Use of Stem Cells: Where Are We?

During the forthcoming years, advances in orthobiologics for sports medicine applications are expected using regenerative technologies such as cellular therapies, gene transfer and tissue engineering [138].

The promotion of healing and regeneration of soft tissue is the main scientific rationale behind using orthobiologics therapies, especially, stem cells. Recently, more attention has been given to multipotent progenitor cells (i.e., embryonic and mesenchymal stem cells (MSCs)), which can be stimulated by endogenous and exogenous factors, obtaining an activation with consecutive differentiation in different cell types, making tendon regeneration and graft incorporation [139], avoiding host reactions and restoring living tissue substitutes [140] possible.

However, in accordance with European and US legislation, these products are defined as “high-risk products” and classified as Advanced Therapy Medicinal Products (ATMPs) [141]; for its use, formal authorisation by a local institutional review board (IRB) is needed. Orthobiologics combines scaffolds, cells stimuli and MSCs to differentiate cells into specialised tissues, such as bone, tendon, cartilage, muscle and ligament, and there is an increased risk for developing teratoma using pluripotent stem cells, namely embryonic stem cells and induced pluripotent stem cells (iPSCs) [142,143].

At present, an ideal cell source selection represents the major challenge in this field due to the lack of specific and standardised isolation protocols, absence of tendon-specific molecular markers and adequate differentiation protocols. Obviously, several difficulties remain about tendon cell populations isolation. Indeed, their use lacks evidence-based approaches: initially, they were isolated following collagenase digestion from a tendon fragment explant from patients [144], but novel methods have been proposed and no consensus between the type of digestion mix and duration was reached [145].

Stem cells in tendon are 1–4% of all nucleated cells and are a heterogeneous cell mix. Implantation of autologous tenocytes is being studied [146] (in phases 2–3, NCT01343836), but it is still not clear whether transplanted cells include only a mixed cell population (as tendon-derived stem cell (TDSCs) and TSPCs) or differentiated cells. In a rat Achilles tendon injury model, TDSCs showed healing abilities [147], highlighting their use to regenerate tendon-like tissue, but their mechanisms are not yet clear.

Regarding MSCs from non-tendon tissues, bone marrow-derived MSCs (BMD-MSCs) are the most evaluated for tissue engineering for regenerative treatment [148,149], and adipose tissue-derived stem cells (ATDSCs) are increasingly used due to their high proliferative properties. BMD-MSCs are obtained after centrifugation, from iliac crest aspiration, and in vitro studies evidenced bi-directional crosstalk between tendon cells and BM-MSCs, inducing tendon-related genes expression (i.e., scleraxis, tenomodulin, collagen type I, decorin and tenascin) with significant ECM deposition [150]. Furthermore, BM-MSCs may survive 8 weeks after injection in rabbit patellar tendon, with described differentiation in tenocyte-like cells in 5 weeks [151]. Despite the limited number of TDSCs which can be obtained in isolation techniques, better regenerative properties have been found compared with BM-MSCs [149], but more studies are needed to define their role and support for healing process.

MSC use has been investigated, showing good results, for rat Achilles tendon injury [152], in the medial collateral ligament (MCL) [153]. Nourissat et al. [154] compared Achilles tendon tear in rats treated with surgery alone (G1), surgery and chondrocyte injection (G2), and surgery and MSC injection (G3), reporting, using a histologic scoring system, better results in G3 showing improvement in enthesis reorganisation, with morphologic and biomechanical aspects similar to a native enthesis. The association of MSCs and collagen injections has been postulated but more studies are needed.

### 3.6. Limitations

One limitation of this narrative review is that the authors did not focus on a single aspect of AT, such as only the pathogenesis or the management; furthermore, our aim was to give readers a comprehensive knowledge of both.

Another limitation is that, given the nature of the present review, we did not perform a systematic search or a meta-analysis of the available scientific literature; however, the information reported were retrieved mainly from high-level articles, thus providing high-quality information to readers.

## 4. Conclusions

AT management remains a major challenge; however, advances in operative management have been made and were supported by several studies regarding the pathologic changes due to overuse in tendon [1].

The natural course and clinical evolution of AT are still unclear, but this condition seemed to be self-limiting for several patients. Lesions present in AT occur when the healing response of the tendon fails, and differences are dependent on the lesion site [1,40]. Therefore, it is of crucial importance to understand if many of the commonly used treatments, including surgery, are really effective [24].

A good practice should be to refer patient to a physical therapist to start a program of EE. But, if patients do not improve after that, then shock wave therapy or nitric oxide patches might be considered as an adjunct, although data on their efficacy are limited [24].

The high recurrence rate for AT when managed conservatively reflects the chronic and recurrent character of this condition [1]. The possibility to undergo a surgical treatment should be discussed with the patient after at least three to six months of nonoperative management [20]. Good outcomes have been obtained in refractory cases in AT with the use of surgery [1]. However, further controlled studies to evaluate and improve more novel treatment approaches are needed [1].

Patients should be aware that symptoms related to AT may recur with either conservative or surgical management. For this reason, they should know how to control their symptoms and when to refer to a clinical specialist through autonomous monitoring, since it may be more beneficial than leading them to believe that AT is fully curable [24].

Regarding MSCs and similar orthobiologic therapies, they can be considered as a novel therapeutic option in the orthopaedic field, with the ability to reconstitute injured tendon tissue and promote tissue healing, but the functional and clinical results need more investigations. Future research must be performed using standardised protocol to obtain cells and to individuate tenogenic differentiation phases. However, interest is rising regarding MSCs and BM-MSCs, and several studies will be published in the forthcoming years. Despite therapeutic optimisation strategies being at an early stage, their impact needs to be critically evaluated in a scientific manner.

As reported in the present review, there is consistent and scientifically sound evidence about the pathogenesis and management of AT; however, more information from further high-quality studies (such as RCTs) are needed especially about the outcomes of conservative treatments (such as physical and injection therapies), since several issues about their efficacy and safety still need to be addressed.

## Data Availability

Not applicable.
